# The association of triglyceride glucose waist-to-height ratio index with depression in United States adults

**DOI:** 10.3389/fnut.2025.1558342

**Published:** 2025-07-02

**Authors:** Rangrang Zhang, Nanfang Li, Delian Zhang, Menghui Wang, Reziya Tuerhong, Qin Luo

**Affiliations:** ^1^Hypertension Center of People’s Hospital of Xinjiang Uygur Autonomous Region, Xinjiang Hypertension Institute, Urumqi, Xinjiang, China; ^2^NHC Key Laboratory of Hypertension Clinical Research, Urumqi, Xinjiang, China; ^3^Key Laboratory of Xinjiang Uygur Autonomous Region, Urumqi, Xinjiang, China; ^4^Xinjiang Clinical Medical Research Center for Hypertension, Urumqi, Xinjiang, China

**Keywords:** triglyceride glucose waist-to-height ratio, depression, triglyceride-glucose, insulin resistance, NHANES

## Abstract

**Objective:**

The purpose of this study is to investigate the relationship between the triglyceride glucose waist-to-height ratio (TyG-WHtR) and depression.

**Methods:**

Data were used from the National Health and Nutrition Examination Survey (NHANES) that was conducted between 2005 and 2018, which included 15,630 eligible people. Based on a Patient Health Questionnaire (PHQ) score of more than 10, the participants were each assigned to one of the two groups: a group of depressed individuals (*n* = 1,347) and a group of non-depressed individuals (*n* = 14,283). To investigate the connection between TyG-WHtR and depression, one-way comparative analyses and multifactorial logistic regression were carried out, and subgroup analyses were also used. To do more research into this connection, quartile grouping was used, and restricted cubic spline (RCS) curves were utilized to evaluate the patterns that emerged in the relationship between TyG-WHtR and depression.

**Results:**

An independent and substantial positive correlation between TyG-WHtR and depression was found by multifactorial logistic regression of the data. In the fully corrected model, TyG-WHtR levels were associated with a higher prevalence of depression (OR = 1.19, 95%:1.09–1.29). Analysis of TyG-WHtR quartiles showed a significant trend in Q4 compared to Q1 (trend *p* < 0.001). There is a linear connection between TyG-WHtR and depression. From the RCS curve, we can see that its threshold is 5.07. From the ROC curve, we know that the predictive value of TyG-WHtR is higher than that of body mass index (BMI). Subgroup analyses indicated significant interactions with diabetes, marital status, education, and BMI.

**Conclusion:**

Depressive symptoms are significantly associated with TyG-WHtR, which is a strong positive correlation. This index may provide useful insights into the diagnosis and treatment of depression as related research continues to advance.

## Introduction

One of the most common mental health conditions, depression is defined by a persistently negative mood and severe impairments in role functioning and quality of life (1). Comorbidities and a higher risk of death are often present in conjunction with it (1). According to information provided by the World Health Organization (WHO) in 2017, the total number of people afflicted by depression worldwide reached 322 million, with a prevalence that was more than 7.5% among women aged 55–74 years (2). It is anticipated that the economic cost of depression will almost quadruple by the year 2030 (3), with an estimated return of $4 for every dollar spent on treatment (4). Depression is a condition that affects between 5 and 10% of the population in the United States; however, it may reach as high as 40% in some groups (5). Even though there are successful therapies, relatively few individuals who are afflicted get sufficient care (6). Depression has a considerable influence on the incidence of comorbid illnesses such as diabetes and is a major risk factor for suicide, with suicide rates rising by 25% over the last 15 years (7). Even though treating and screening for depression may be difficult, the role of depression in clinical practice continues to be of the utmost importance.

Insulin resistance (IR) is a metabolic pro-inflammatory condition that may be modified and affects approximately one-third of individuals who seem to be in good health. IR is defined as the diminished responsiveness of insulin-target tissues to normal physiological insulin levels, which ultimately results in higher peripheral blood glucose levels. Inflammation is recognized to play a vital role in the development of many chronic illnesses (8, 9). A bidirectional relationship between IR and depression has been hypothesized, where IR not only increases the risk of developing depression but may also exacerbate its severity. Conversely, depression can heighten the risk of developing IR due to shared inflammatory pathways and other common mechanisms (9).

The triglyceride-glucose index (TyG index) is a reliable tool for assessing insulin resistance to insulin (10, 11). Several diseases and clinical outcomes, including depression, have been shown to correlate with this score (10, 11). Most of the previous studies have focused on TyG and indicators such as TyG-WC, TyG-BMI, and WHtR. Although triglyceride glucose waist-to-height ratio (TyG-WHtR) is a new TyG-modified index that has not been thoroughly investigated previously, this index has recently become a hot topic of research and has been shown to be strongly associated with a variety of diseases, such as cardiovascular disease, psoriasis, metabolism, gallstones, and periodontitis (12–16). However, the link between TyG-WHtR and depression has not been fully elucidated by research. Therefore, this study aimed to investigate the relationship between TyG-WHtR and depression. Using information obtained from the NHANES between 2005 and 2018, this relationship between the TyG-WHtR index and depression is the subject of this study, which aims to analyze the connection between the two.

## Methods

### Subjects and study design

**Figure 1:**
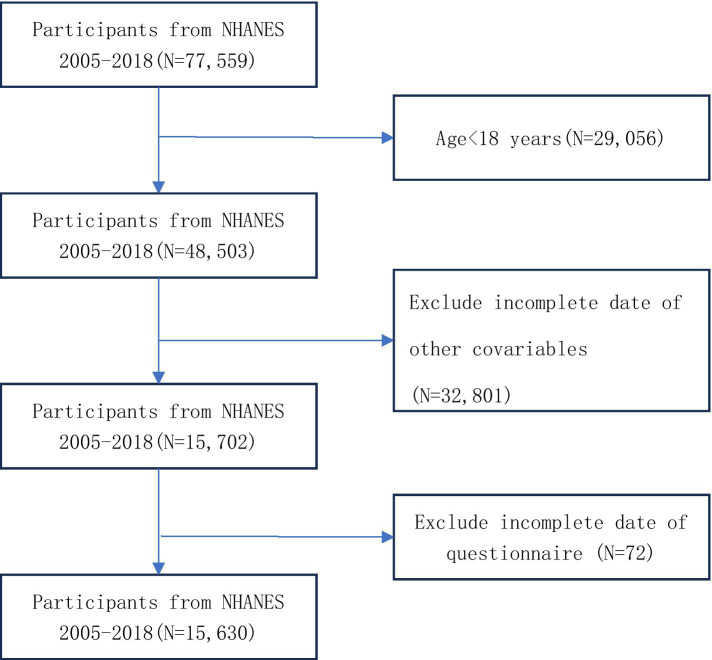
Study population selection (*N* = 15,630).

This study analyzed data from 7 cycles of the NHANES, comprising 15,630 participants aged 18 years or older after data cleaning. The analysis utilized demographic information, laboratory test results, disease history from questionnaires, and body measurements. Participants were excluded based on the following criteria: (1) missing data for glucose, height, waist circumference, or triglycerides; (2) missing depression scale data; (3) missing data for other covariates; and (4) age under 18 years. Detailed information on participant selection is provided in Figure 1.

### Definitions and the collection of data

Drawn from the existing body of literature (17, 18), according to the findings of this research, several factors have the potential to confuse the connection between the TyG-WHtR index and depressed symptoms. In addition to gender, age, the poverty impact ratio (PIR), smoking, alcohol use, race, hypertension, diabetes, body mass index (BMI), and education level, these factors also include diabetes and hypertension. According to the definition, hypertension may be defined as the following: (1) a systolic blood pressure of 140 mmHg or higher or diastolic blood pressure of 90 mmHg or higher, measured for 3 days that are not consecutive; (2) a diagnosis from a physician; and (3) the current use of antihypertensive medication. If any of the following criteria were met, a diagnosis of diabetes mellitus was made: (1) a glycosylated hemoglobin (HbA1c) level of 6.5% or above, (2) a fasting blood glucose level of 126 mg/dL or higher, (3) a diagnosis from a medical professional, or (4) the current use of insulin or other hypoglycemic drugs. Mexican American, non-Hispanic White, non-Hispanic Black, and others were the categories that were used to classify one’s race. PIR was divided into three categories: <1, 1–3, and >3, depending on the replies received from the questionnaire. The levels of educational attainment were classified as lower than high school, high school, and higher than high school. Each individual’s marital status was classified as either married or unmarried. To be considered a smoker, someone must have smoked a minimum of 100 cigarettes throughout their lifetime. On the other hand, alcohol drinkers are classified as those who consume more than 12 drinks in a single year. The BMI was classified into three distinct categories, namely “<25 kg/m^2^,” “25 ~ 30 kg/m^2^,” and “>30 kg/m^2^.” Congestive heart failure, coronary heart disease, cancer or malignancy, liver illness, and diabetes were some of the other comorbid disorders that were taken into consideration, according to the diagnoses provided by different physicians. The following formula was used to determine the TyG-WHtR index: The formula for calculating the TyG-WHtR is as follows: TyG-WHtR = [ln(Fasting Triglycerides (mg/dl) × Fasting Glucose (mg/dl)/2)] × (Waist Circumference (cm) / Height (cm)) (19). The PHQ-9 questionnaire was used to evaluate the presence of depressive symptoms, and individuals were deemed to be depressed if their score on the PHQ-9 was equal to or more than 10.

### Statistical analysis

Whether or not the individuals were exhibiting signs of depression, they were separated into different groups. Means and standard errors were determined for continuous variables, whereas categorical variables were analyzed using frequencies and percentages. Methods, such as the chi-square test and the Kruskal–Wallis *H*-test, were used to analyze the differences that existed between groups that had different TyG-WHtR quartiles. Restricted cubic spline (RCS) curves were used to analyze the non-linear relationship between TyG-WHtR and depression and determine the cutoff value (we first used the *rms* (Frank Harrell, Vanderbilt Univ, USA) package to perform RCS regression analysis on the relationship between the variable TyG-WHtR and depression, testing models with 3, 4, and 5 nodes (nodes), and selected the “four-node” model based on the Akaike Information Criterion (AIC) for better fit and explanatory power). We then visualized the regression results using the *Predict()* (included in rms) function and the *ggplot2* (Hadley Wickham, RStudio/Posit, USA) package, generating a curve showing the variation in TyG-WHtR and OR values. The *Predict()* function established a one-to-one correspondence between TyG-WHtR values and OR values. We selected the TyG-WHtR value at OR = 1 as the threshold, which was 5.07, at which point the slope of the curve changed significantly, representing the “cutoff value.” This is a common exploratory method in epidemiological research. When analyzing the relationships between TyG-WHtR and depression, logistic regression was the method of choice. To control for possible confounding factors, three models were developed: The first model is unadjusted, the second model is adjusted for education, personal income, marital status, and ethnicity, and the third model is further adjusted for covariates related to congestive heart failure, body mass index (BMI), smoking, alcohol use, coronary artery disease, liver disease, cancer or malignant neoplasm, and diabetes mellitus, beyond the adjustments that were made in the second model.

## Results

### Baseline characteristics

**Figure 2:**
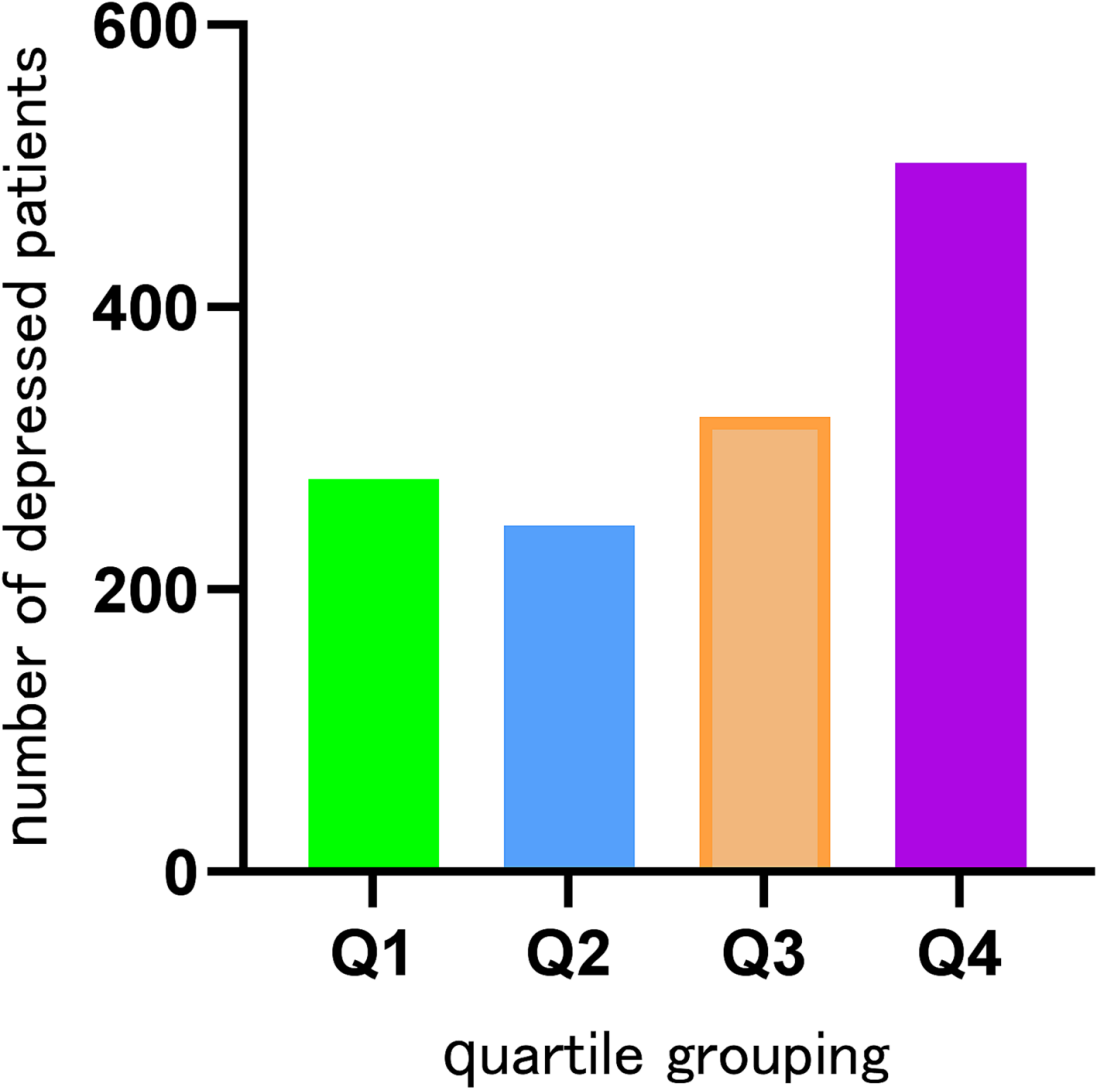
Number of depressions in each of the quartile subgroups.

This research was conducted with the participation of 15,630 individuals who were drawn from the NHANES database. Based on whether or not they suffered from depression, the participants were divided into two distinct groups: 1347 individuals who were depressed and 14,283 individuals who did not suffer from depression. The highest percentage of Q4 tertile numbers was found in depressed patients (Figure 2). Of the study population, 7,737 were male (49.5%) and 7,893 were female (50.5%). Among the population of women, the prevalence of depression was found to be the greatest, at 63.1%. Smokers had a higher likelihood of depression, with a prevalence of 60.7%. Individuals with a high school education or higher had the highest depression rate at 40.2%. Additionally, non-Hispanic White individuals (44.6%), those with a BMI > 30 (46.8%), unmarried individuals (64.7%), and those with a PIR between 1 and 3 (44.1%) exhibited higher depression prevalence. The baseline characteristics of the research population are presented in great depth depending on whether or not they were depressed, as shown in Table 1. When comparing age, cancer, or race to depression, there were no statistically significant differences found (*p* > 0.05). However, TyG-WHtR, gender, congestive heart failure, education level, coronary heart disease, marital status, liver disease, alcohol, smoking, hypertension, PIR, BMI, and diabetes mellitus showed correlations with depression that are statistically significant (*p* < 0.05) (Table 1).

**Table 1:** Characteristics of the study population between TyG-WHtR and depression (*N* = 15,630).

Characteristic	Non-depression (*n* = 14,283)	Depression (*n* = 1,347)	*p*-value
Age	49 (34.00, 63.00)	50 (37.00, 61.00)	0.664
Gender			<0.001
Male	50.7%	36.9%	
Female	49.3%	63.1%	
Smoking habit			<0.001
Yes	44.8%	60.7%	
No	55.2%	39.3%	
Drinking status			0.022
Yes	74.6%	71.8%	
No	25.4%	28.2%	
Coronary heart disease			<0.001
Yes	3.8%	4.0%	
No	96.2%	96.0%	
Congestive heart failure			<0.001
Yes	2.6%	5.8%	
No	97.4%	94.2%	
Cancer or malignancy			0.312
Yes	9.1%	9.9%	
No	90.9%	90.1%	
Hepatic disease			<0.001
Yes	3.5%	7.3%	
No	96.5%	92.7%	
Diabetes			<0.001
Yes	17.3%	24.7%	
No	82.7%	75.3%	
Hypertension			<0.001
Yes	34.8%	46.2%	
No	65.2%	53.8%	
PIR			<0.001
≤1	18.9%	39.8%	
1–3	41.8%	44.1%	
>3	39.3%	16.1%	
Education			<0.001
Under high school	22.7%	35.7%	
High school	22.8%	24.1%	
Higher than high school	49.8%	40.2%	
Race			0.232
Mexican American	15.9%	16.0%	
Non-Hispanic White	46.6%	44.6%	
Non-Hispanic Black	19.0%	21.2%	
Other	18.5%	18.2%	
BMI, kg/m^2^			<0.001
<25	29.2%	26.7%	
25–30	34.5%	26.5%	
>30	36.3%	46.8%	
Marriage status			<0.001
Married	54.0%	35.3%	
Unmarried	46.0%	64.7%	
TyG-WHtR	5.05 (4.37, 5.77)	5.41 (4.57, 6.27)	<0.001

PIR, poverty impact ratio; TyG-WHtR, triglyceride glucose waist-to-height ratio.

### TyG-WHtR and depression

**Figure 3:**
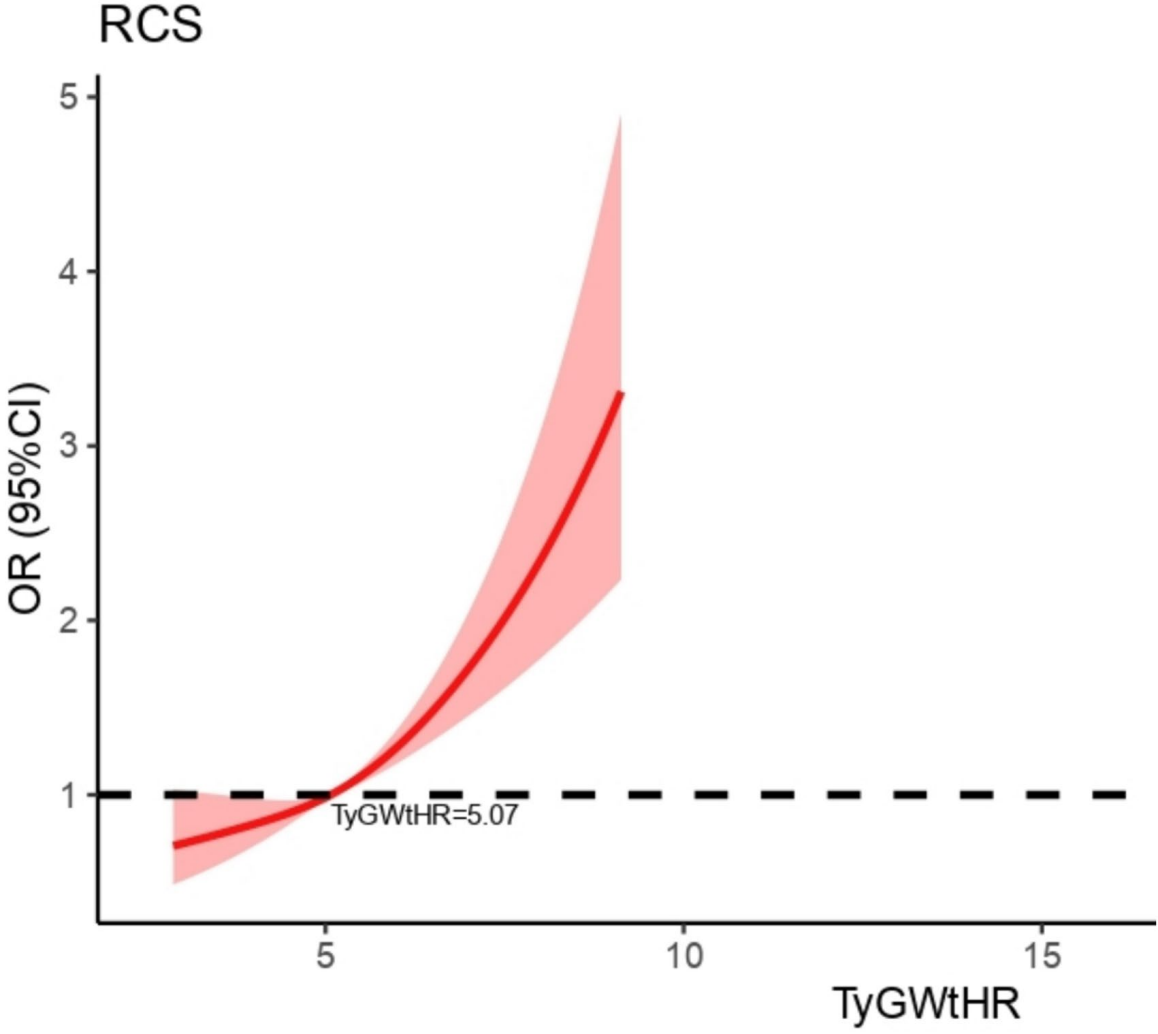
Dose–response relationship between TyG-WHtR and depression.

**Figure 4:**
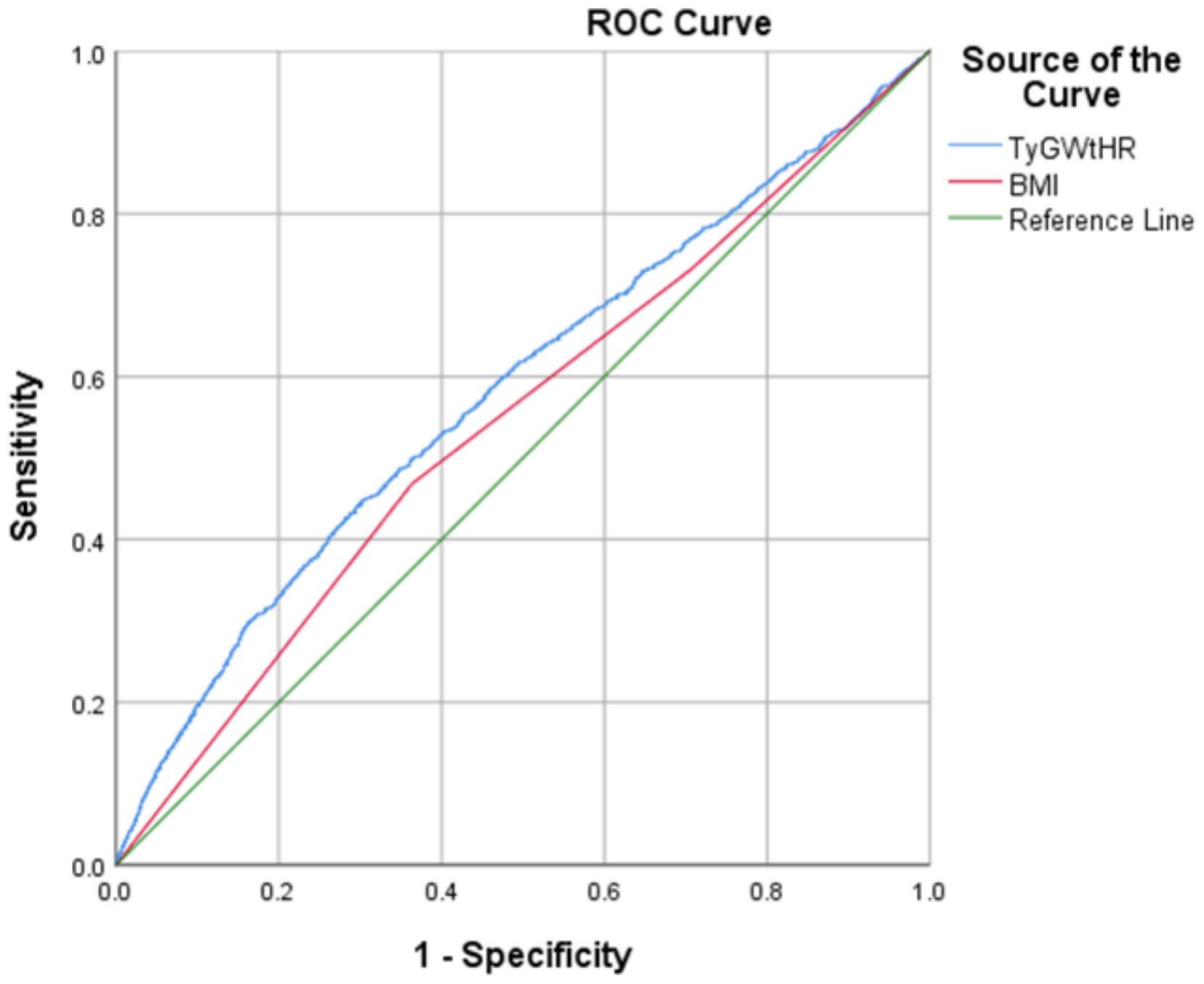
Comparison of the predictive value of TyG-WHtR and BMI.

As shown in Table 2, the multifactorial logistic regression analysis models that investigate the connection between TyG-WHtR and depression are presented. In each of the three models, the continuous variable TyG-WHtR had a positive connection with hypertension. The odds ratios (ORs) for each of the three models were 1.34 (95% CI: 1.27, 1.40), 1.28 (95% CI: 1.22, 1.35), and 1.19 (95% CI, 1.09, 1.29), respectively. In the fully corrected model, TyG-WHtR levels were associated with a higher prevalence of depression (OR = 1.19, 95%:1.09–1.29). Analysis of TyG-WHtR quartiles showed a significant trend in Q4 compared to Q1 (trend *p* < 0.001). This was the case when the continuous variable TyG-WHtR was turned into a categorical variable. The limited cubic spline curve provided more evidence of the considerable positive correlation that exists between TyG-WHtR and depression. In addition, through the RCS curve (which can be used to find cut points), we can see that its inflection point is 5.07, and when TyG-WHtR exceeds this value, the depressed group shows a significant positive correlation with an OR value >1 compared to the non-depressed group. At this point, it may be necessary to be alert to the possibility of depression. When the TyG-WHtR value is below this threshold, its OR is <1. Additionally, the curve demonstrated that there is a linear link between the two variables. The results of the ROC curve showed that the area under the ROC curve for TyG-WHtR in predicting depression was 0.583, higher than the area under the curve (AUC) of 0.545 for BMI (Tables 2, 3; Figures 3, 4).

**Table 2:** Association between TyG-WHtR and depression.

Expose	Model 1	Model 2	Model 3
	OR (95%CI) *P*-value	OR (95%CI) *P*-value	OR (95%CI) *P*-value
TyG-WHtR	1.34 (1.27, 1.40) < 0.0001	1.28 (1.22, 1.35) < 0.0001	1.19 (1.09, 1.29) < 0.0001
TyG-WHtR index quartile			
Q1	1.00	1.00	1.00
Q2	0.87 (0.73, 1.04) 0.135	0.91 (0.76, 1.09) 0.317	0.93 (0.75, 1.14) 0.457
Q3	1.17 (0.992, 1.39) 0.061	1.17 (0.986, 1.39) 0.072	1.12 (0.88, 1.41) 0.365
Q4	1.93 (1.65, 2.25) < 0.0001	1.77 (1.51, 2.08) < 0.0001	1.37 (1.05, 1.79) 0.020
*p* -for trend	<0.001	<0.001	0.003

TyG-WHtR, triglyceride glucose-waist-to-height ratio.

Model 1: unadjusted; Model 2: education, PIR, marital status, and race; Model 3: education, gender, drinking status, smoking habit, coronary heart disease, congestive heart failure, hepatic disease, diabetes, hypertension, BMI, PIR, marriage status.

**Table 3:** Results of multifactor logistic regression analysis.

Variable	Odds ratio	95%CI	*p*-value
Gender	0.56	0.50–0.64	<0.001
Alcohol	0.99	0.86–1.14	0.891
Smoking	1.85	1.63–2.09	<0.001
DM	1.10	0.95–1.29	0.215
PIR			<0.001
PIR-(1)	1.00		
PIR-(2)	0.59	0.51–0.67	<0.001
PIR-(3)	0.29	0.24–0.35	<0.001
Education			0.002
Education-(1)	1.00		
Education-(2)	0.81	0.69–0.95	0.008
Education-(3)	0.78	0.68–0.90	0.001
Hypertension	1.27	1.12–1.45	<0.001
BMI			<0.001
BMI-(1)	1.00		
BMI-(2)	0.71	0.60–0.84	<0.001
BMI-(3)	0.86	0.70–1.06	0.163
Marriage	0.62	0.55–0.70	<0.001
TyG-WHtR	1.19	1.09–1.29	<0.001
TyG-WHtR quartile			0.005
Q1	1.00		
Q2	0.93	0.75–1.14	0.457
Q3	1.12	0.88–1.41	0.365
Q4	1.37	1.05–1.79	0.020
Coronary heart disease	1.28	0.98–1.69	0.076
Congestive heart failure	1.39	1.05–1.84	0.022
Hepatic disease	1.80	1.42–2.28	<0.001

### Analysis of subgroups

**Figure 5:**
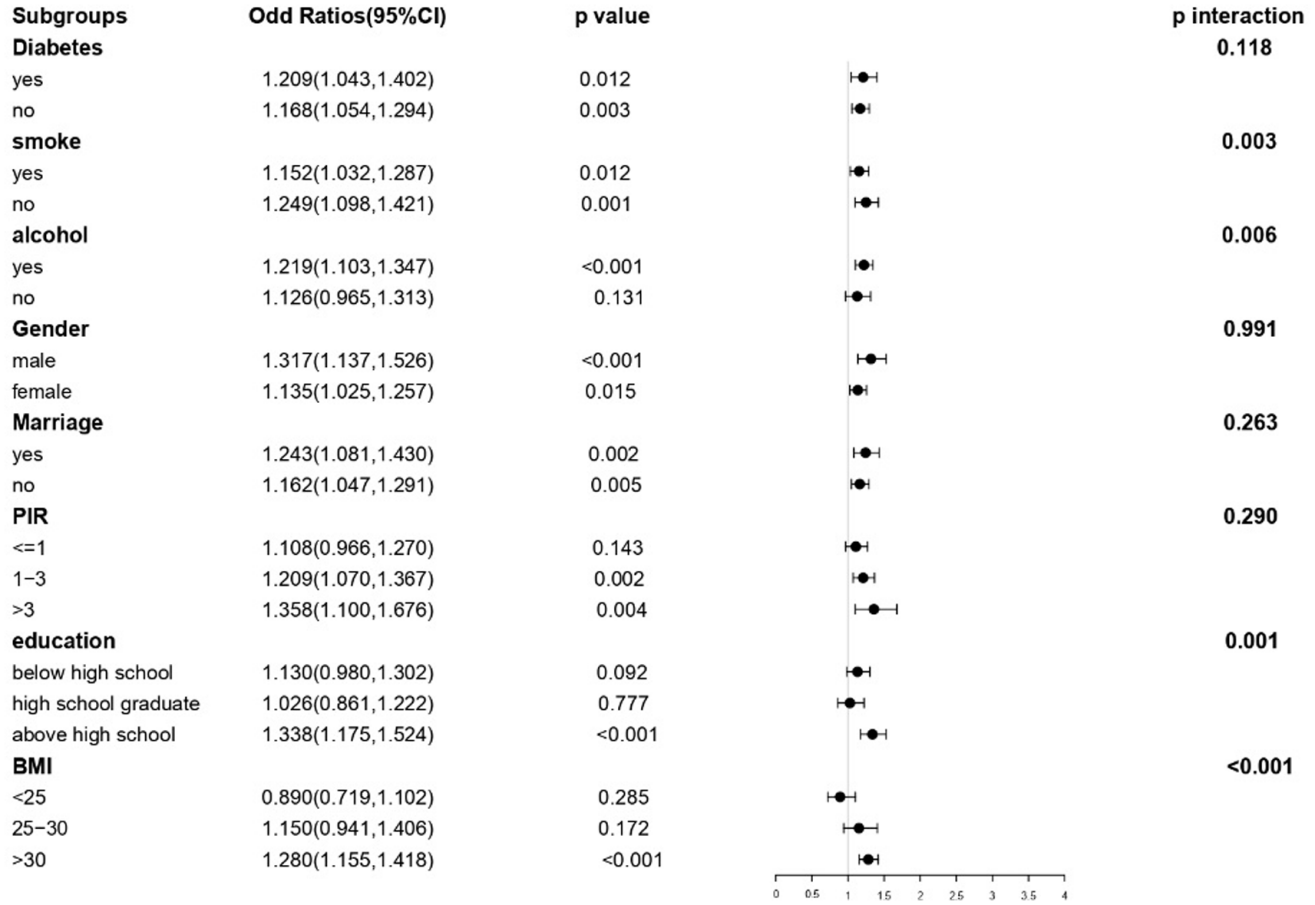
Subgroup analysis of the association between TyG-WHtR and risk of depression.

To further examine the possible correlation between TyG-WHtR and depression under varying situations, stratified analyses were performed, including gender, diabetes status, and other pertinent characteristics. A significant correlation between TyG-WHtR and depression was observed in subgroups stratified by diabetes, sex, marital status, and smoking. Greater BMI, higher education, greater PIR, married status, male gender, alcohol consumption, diabetes, and married status groups had greater ORs. In contrast, no significant correlation was found in subgroups of non-alcohol, individuals with PIR ≤ 1, those with a high school education or lower, and those with a BMI of <30. Furthermore, the subgroup analysis demonstrated that there were noteworthy interactions between TyG-WHtR and elements such as smoking (*p* = 0.003), drinking (*p* = 0.006), education level (*p* = 0.001), and body mass index (BMI) (*p* < 0.001) (Figure 5).

## Discussion

This study examined the link between the TyG-WHtR index and depression in the adult population of the United States. This research found that elevated TyG-WHtR levels correlated with a higher prevalence of depression. Additionally, it was found that there was a linear connection, with the point of inflection occurring at a value of 5.07 for the TyG-WHtR. A 19% increase in the risk of depression was related to each one-unit rise in TyG-WHtR that occurred after this threshold was reached. Subgroup analyses identified significant interactions between TyG-WHtR and diabetes, drinking, education level, and BMI, whereas no interactions were observed with diabetes, gender, PIR, or marriage status. This study’s findings provide significant new insights into the relationship between TyG-WHtR and depression. On the other hand, further study is required to analyze the possible pathways implicated and to understand the underlying processes that are responsible for this link.

According to the findings of the Global Burden of Disease 2019 report, mental illnesses continue to be among the top 10 global health burdens, with the incidence of depression having dramatically increased over the last century. It is estimated that approximately 280 million individuals throughout the globe are now suffering from depression (20). According to statistics provided by the World Health Organization (WHO), 5% of individuals have symptoms of depression. The worldwide health epidemic of depression, which often manifests itself in younger populations, continues to be a neglected issue, even though it is ubiquitous (21). Among the primary causes of disability, depression is also a significant contributor to the overall burden of illness across the world (22). Approximately 50% of all psychiatric consultations and 12% of all hospitalizations are attributed to it, making it a significant factor in determining both quality of life and survival (23). Furthermore, research has shown that there is a significant connection between depression and chronic physical disorders. A decline in physical health has a detrimental influence on mental health, and vice versa is the case (24, 25). As such, early detection and diagnosis of depression are essential for effective recovery.

It has been established that diabetes and depression share a significant bidirectional relationship, with the occurrence of one condition tripling the risk of the other (26). Within the scope of this discussion, insulin resistance, a characteristic feature of diabetes, is directly linked to the development and pathophysiology of depression (27). Despite the existence of diabetes, it is interesting to note that insulin resistance (IR) has been recognized as a risk factor for depression on its own. For example, Lee et al. revealed in cross-sectional research that higher IR was associated with the development of depression in the general Korean population. This was shown by observing the correlation between the two variables (27). In addition, previous studies have explored the association between peripheral lipids, depression, and cognition. In the Family Risk Study, lipid-associated medical disorders (LAMDs) were found to be associated with depression and cognitive outcomes, and LAMDs increased the risk of depression. In the NHANES study, HDL levels were positively associated with cognition, but this association was moderated by depressive states, and depression diminished the protective effect of High-Density Lipoprotein (HDL) on cognition. The study suggests that lipid dysregulation may be a common underlying biological mechanism for depression and cognitive changes (28).

Several metabolic markers have been linked to depressed symptoms, including BMI, waist circumference, and the TyG waist circumference, according to other research (29). As a realistic surrogate marker for IR, the TyG index has been devised, and it has shown both excellent sensitivity and specificity (30, 31). There have also been connections made between the TyG index and several illnesses, such as diabetes, cardiovascular disease, and cognitive dysfunction (18, 32–35). There is a significant amount of use of the TyG index as a biomarker to assess IR in clinical settings. Particularly helpful in the diagnosis and treatment of conditions such as cardiovascular failure, diabetes, high blood pressure, and metabolic problems, which are ailments that may be diagnosed and treated with this substance (32, 36, 37). The TyG index is both simple and inexpensive to obtain (32, 36, 37). It is important to note that nationwide retrospective cohort research that included 8,287 individuals aged 45 and older discovered a positive link between the TyG index and the development of depression (38). This correlation was stable across numerous sensitivity analyses (38).

Recently, the combined use of various indices has become a prominent research focus, with the TyG-WHtR index standing out as a modification of the TyG index, incorporating the waist-to-height ratio. The TyG-WHtR was shown to be independently related to an elevated risk of cardiovascular disease in Chinese populations who were middle-aged or elderly, according to research that was conducted in China using a national cohort. When the researchers took into account several possible confounding factors, they discovered that higher cumulative TyG-WHtR levels were associated with a higher risk of cardiovascular events (OR: 1.27, 95% confidence interval: 1.12–1.43) (19). Moreover, this score has been linked to a greater prevalence of gallstones in the population (39). However, its relationship with depression has been less explored. A substantial positive and linear correlation was established between TyG-WHtR and depression for this research, which intended to evaluate the link between the two. The identified cutoff point was 5.07, beyond which the risk of depression increased significantly. Subgroup analysis further confirmed the stability of this correlation.

There is a lack of complete comprehension of the specific mechanism that underlies the correlation between the TyG index and depression, which calls for more research. However, several potential mechanisms have emerged in the current study. The most likely mechanism involves the association of the TyG index with IR. We showed that IR plays a significant role in the link between the TyG index and depression (OR = 1.17, 95%CI:1.07–1.27), which suggests that IR is a risk factor for depression (27). This was discovered by large-scale Korean research that included 165,443 individuals (27). The TyG index was shown to be a viable marker for measuring IR, according to a meta-analysis conducted by Nabipoorashrafi et al. (40). This index exhibited significant concordance with the usual gold standard for IR evaluation (40). Moreover, numerous basic studies have established a robust bidirectional relationship between depression and physical health, particularly concerning diabetes and IR (40). Research indicates that IR, while a hallmark of diabetes, may also independently contribute to depression (27). Specifically, IR may influence the development of depression by disrupting neurotransmitter homeostasis in the brain, triggering inflammatory responses, and impairing nerve growth factor function (40). Additionally, the negative impact of chronic physical illnesses may exacerbate mental health issues, further contributing to the onset of depression (41). This interplay between physical and mental health underscores the complexity of the relationship between IR and depression (41). It has been shown that there is a correlation between the TyG index and inflammatory markers (42). Inflammation may cause damage to the endothelium of the blood vessels and promote oxidative stress, both of which are factors that contribute to illnesses such as vascular depression and vascular cognitive impairment (43). The ROC curve suggested an AUC value of 0.583, a relatively small value. We consider that it may be related to the following reasons: (1) the sample of individuals in our study was heterogeneous and varied widely in number; (2) as a cross-sectional study, the cause-and-effect link between metabolic disorders and depression cannot be established with any degree of certainty; (3) indicator characteristics: TyG-WHtR integrates two dimensions, insulin resistance and central obesity, but there may be differences in the mechanism of the effect of the two on depression; (4) measurement error: waist circumference measurement is affected by respiratory status, and there is a significant intraday triglyceride level variation; and (5) population heterogeneity: the high prevalence of metabolic disorders in the US population may have weakened the discriminatory power of the indicators. Nonetheless, our study found an association between this composite metabolic indicator and depression in a representative US population, providing new insights for interdisciplinary metabolic-mental health research.

The study data were obtained from the NHANES dataset, and a standardized sampling method was used to include a large sample size to ensure reliability and representativeness of the data. The results of the study may provide new insights into the prevention and treatment of depression.

Despite controlling for key demographic and other confounders, there may be unmeasured confounding variables that affect the results, such as genetic susceptibility, objective physical activity data, and key indicators such as sleep quality. These factors may affect both metabolic indicators and depression risk, for example, sleep disorders may contribute to both insulin resistance and affect mood regulation. Second, due to the inherent limitations of cross-sectional designs, we cannot rule out the possibility of reverse causality. Depressive symptoms themselves may lead to metabolic abnormalities through both behavioral (e.g., emotional eating of high-fat and high-sugar foods, decreased activity) and biological [e.g., chronic inflammatory state, Hypothalamic-Pituitary-Adrenal (HPA) axis dysfunction] mechanisms. Together, these factors may have contributed to the more limited predictive efficacy (AUC = 0.583) observed in this study. Further prospective studies on TyG-WHtR and depression are needed in the future.

## Conclusion

The incidence of depression was shown to have a strong positive association with an increase in the TyG-WHtR index, even after taking into account the potential influence of other factors. To have a better knowledge of the relationship between insulin resistance and the likelihood of experiencing depression, this research offers a significant foundation.

## Data Availability

The original contributions presented in the study are included in the article/supplementary material, further inquiries can be directed to the corresponding author.
